# Association between epidural analgesia and postpartum psychiatric disorders: A meta-analysis

**DOI:** 10.1016/j.heliyon.2024.e27717

**Published:** 2024-03-08

**Authors:** Solmaz Ghanbari-Homaie, Seyedeh-Pooneh Jenani, Leili Faraji-Gavgani, Pooya Hosenzadeh, Mansour Rezaei

**Affiliations:** a Department of Midwifery, Faculty of Nursing and Midwifery, Tabriz University of Medical Sciences, Tabriz, Iran; b Academic Board Member, Department of Midwifery, Islamic Azad University of Medical Science, Marand, Tabriz, Iran; c Research Center for Evidence Based Medicine, Tabriz University of Medical Sciences, Tabriz, Iran; d Clinical Research Development Unit of Taleghani Hospital, Tabriz University of Medical Sciences, Tabriz, Iran; e Department of Anesthesiology, School of Medicine, Tabriz University of Medical Sciences, Tabriz, Iran

**Keywords:** Birth, Analgesia, Mental health, Postpartum depression, Anxiety, Post-traumatic stress disorder, Systematic review

## Abstract

**Aim:**

Women during the postpartum period are at risk for psychiatric disorders such as postpartum depression (PPD), post-traumatic stress disorder (PTSD), and anxiety. It is controversial whether labour epidural analgesia have a protective role on PPD, PTSD, and anxiety or not. This study is a meta-analysis of previously published observational studies to investigate the association between epidural analgesia and PPD, PTSD, and anxiety.

**Methods:**

We searched Cochran Library, PubMed, ProQuest, EMBASE, Scopus, Web of Science, PsycINFO, and MEDLINE from inception until December 2022. The outcome measures were positive screen of PPD, PTSD, and anxiety among women who received labour epidural analgesia. The Newcastle-Ottawa Scale was used to assess the quality of the observational studies. Statistical analysis was performed using Stata 17.0 software. The mean differences or odds ratios were pooled based on random effect.

**Results:**

We included 31 studies (12,064 women) in the review. The meta-analysis of PPD studies reported mean (standard deviation) showed no significant association between epidural analgesia and PPD (Mean Difference = 0.01; 95% Confidence Interval = −0.14 to 0.16; p-value = 0.88). The meta-analysis of PPD studies reported percentage showed no statistically significant difference in terms of the chance of depression between the exposed and non-exposed groups (Log Odds Ratio = −0.61; 95% CI = −1.48 to 0.26; p-value = 0.17). The meta-analysis of PPD studies reported OR showed indicate a lower chance of depression in the epidural group compared to the non-epidural group (Effect size = 1.01; 95% CI = 0.64 to 1.38; P-value = 0.00). In the case of PTSD, due to the heterogeneity of the tools and the methodology of the studies, it was impossible to reach a definitive conclusion and measure the studies in a meta-analysis. The meta-analysis of anxiety studies showed that no significant association between epidural analgesia and anxiety (MD = −1.36; 95% CI = −3.38 to 1.14; p-value = 0.29).

**Conclusion:**

Based on the meta-analysis of observational studies, epidural analgesia for labour pain relief has no protective effect on postpartum psychiatric disorders.

## 1 Introduction

Birth is a women's most challenging physiological and psychological experience [1]. Women during the postpartum period are at risk for psychiatric disorders such as postpartum depression (PPD), post-traumatic stress disorder (PTSD), and anxiety [2].

The most common postpartum psychiatric disorder is postpartum depression, which onset the first month following birth [3]. Studies have shown that postpartum depression affects a significant portion of the global population, with reported rates ranging from 8% to 26% [4,5]. PPD can cause physical and psychological complications for mother and infant [6]. Post-traumatic stress disorder has been defined as “the complex somatic, cognitive, affective, and behavioral effects of psychological trauma” [7]. The prevalence of PTSD following birth is 7.2% [7,8]. Postpartum anxiety has been reported in 16.2% of women within six weeks following birth [9] and may coincide with PPD [10].

The etiology of postpartum psychiatric disorders is complex, and several factors have been identified as its predictors. The intensity of labour pain has been established as a potential predictor for the onset of these disorders [7,11]. It is concluded that labour pain may cause severe emotional states, including anxiety, stress, and fear [12]. Women who experience severe pain during labour release a high amount of catecholamines, which prevents the contraction of the uterus and the progress of labour. This increases women's anxiety and creates a vicious cycle of poor progress of labour, releases more catecholamins, causes more anxiety and other mental illnesses [13]. Depression and chronic pain occur simultaneously in more than 80% of patients. A number of common neural substrates including neurotransmitters, neurotrophins, inflammatory mediators and neuroendocrine alterations are involved in these conditions. Any or all of them may also alter neural function in key brain regions responsible for regulating emotional and pain processing [14]. Therefore, pain relief during labour may improve maternal well-being and enhance maternal and neonatal outcomes, particularly those pertaining to the mother's mental health in the postpartum period [15,16]. A multitude of labour pain management methods have been devised, encompassing both non-invasive and invasive techniques. These include epidural anesthesia [17], inhalation of nitrogen oxide [18], and administration of opioids [19]. According to previous studies, epidural administration is widely regarded as the most effective method for pain management [20].

Regarding the association between pain and psychiatric disorders, several studies assessed the effects of epidural administration on lowering the incidence of PPD, PTSD, and anxiety in women during postpartum [[21], [22], [23]]. However, the findings of these studies are inconsistent [[24], [25], [26], [27]]. Therefore, the clinical issue of whether epidural analgesia can effectively prevent psychological outcomes by decreasing labour pain remains unsolved [28]. This study is a meta-analysis of recently published observational studies investigating the association between epidural analgesia and psychiatric disorders such as PPD, PTSD, and anxiety.

## 2 Methods

Methods were aligned with the Preferred Reporting Items for Systematic Reviews and Meta-Analysis (PRISMA) guideline.

### 2.1 Search strategy and study selection

Eight electronic databases (Cochran Library, PubMed, ProQuest, EMBASE, Scopus, Web of Science, PsycINFO, and MEDLINE) were systematically searched from inception until December 2022. Grey literature was searched via Google Scholar, ResearchGate, and various websites for dissertations. Reference lists of included studies and reviews were explored for further studies. A search strategy was developed using the keywords “Epidural analgesia”, “Postpartum Depression”, “PPD”, “Post-Traumatic Syndrome Disorders”, “PTSD”, “Anxiety”, and “Stress”. SPJ and LFG performed the screening process and independently determined eligibility by reviewing the relevance of all study titles and abstracts. Regarding the lack of sufficient information in the title and abstract, the full text was read to decide whether to include or exclude the study. Disagreements between the two reviewers were resolved by a discussion involving a third reviewer (SGH).

### 2.2 Eligibility criteria

Inclusion criteria were as follows: observational studies (prospective or retrospective longitudinal, cross-sectional) in English investigating the association between epidural analgesia and postpartum psychiatric disorders, including PPD, PTSD, and anxiety. Standard scales were employed to measure the outcomes between one to 24 months postpartum. Standard scales included for PPD: EDPS, Stein's MB scale; For PTSD: PPQ, PC-PTSD, CBiTS, TES and IES; and for anxiety: STAI, PSS. Studies involving women who had an elective cesarean section were excluded.

### 2.3 Data extraction

Data were extracted into a data extraction form in Microsoft Excel by second (SPJ) and third authors (LFG). Data extraction for the included studies were: a) author, b) year of publication, c) country, d) study design, e) the number of participants, f) type of psychiatric disorders, j) final results of the studies (results reported in percentage or mean and standard deviation or odds ratio for case and control group).

### 2.4 Quality assessment

The Newcastle-Ottawa Scale was used to assess the quality of the observational studies included. This scale contains eight items within three domains and a total maximum score of nine. The final quality scores were categorized as follows: “low quality with very high risk of bias” (score< 3); “intermediate quality or intermediate risk of bias” (score 4–6); and “high quality” (score 7–9) [29]. The quality of the included studies was independently assessed by the first author (LFG) and second author (SPJ). In the next stage, judgments were compared, disagreements among the reviewers were finalized by consultation with the first author (SGH), and the final result was obtained.

### 2.5 Data synthesis

All analyses were performed using STATA version 17.0 (Stata Corporation, College Station, TX, USA). The percentage, mean, and odds ratio of risk for psychiatric disorders such as PPD, PTSD, and anxiety were examined. The presence of heterogeneity was assessed using Cochran's Q-test, with a significance level of p < 0.05. The I-square test was also used to calculate the percentage of heterogeneity [30]. The odds ratio (percentage) or mean (standard deviation) were extracted from cohort and case-control or cross-sectional studies. A random-effect model was used to estimate pooled effect sizes. Predefined subgroup analyses were conducted to explore the origin of heterogeneity, with a focus on the sample size, follow-up period, and baseline value for psychiatric disorders. Publication bias was analyzed using funnel plot analysis and Egger's regression asymmetry test [31]**.**

## 3 Results

### 3.1 Search results

**Fig. 1:**
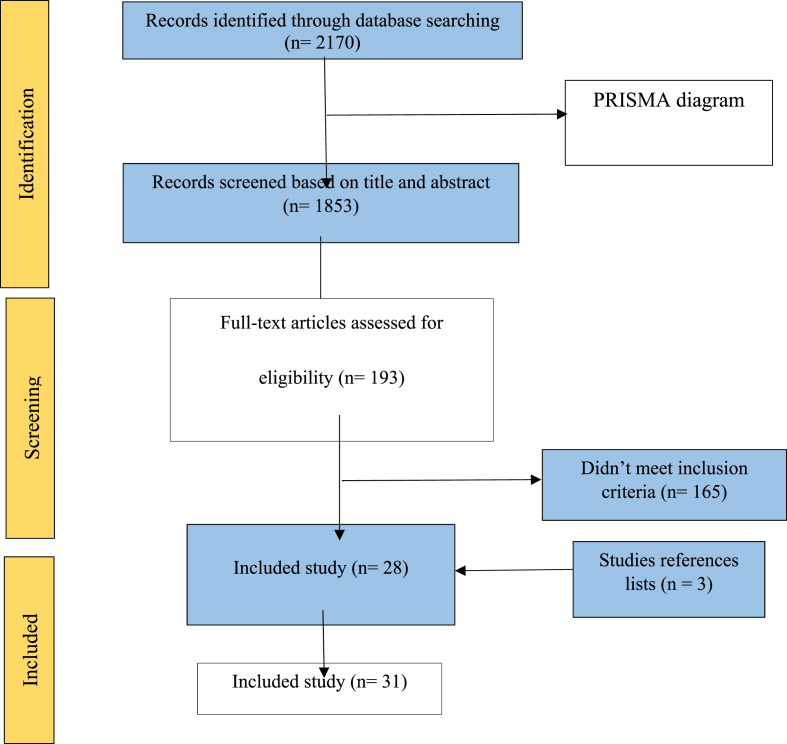
PRISMA diagram for association between epidural analgesia and postpartum psychiatric disorders.

A total of 2170 studies were found in the initial search and entered into the Endnote software. Of these 124 duplicate articles were removed. Leaving 1853 studies were screened based on title and abstract. Of these, 165 studies were excluded because they did not meet the inclusion criteria. Finally, 31 eligible studies were included in the review (Fig. 1). Of these, three studies were cross-sectional [7,23,32], one study was case-control [21], and 27 studies were cohort studies [22,[24], [25], [26], [27],[33], [34], [35], [36], [37], [38], [39], [40], [41], [42], [43], [44], [45], [46], [47], [48], [49], [50], [51], [52], [53], [54]]. The characteristics of the included studies have been summarized in Table 1. The studies were published between 1995 and 2022. The sample size of participants varied from 48 to 80606. The studies were conducted in Australia [45], Canada [26,40,42], China [34,44,50,54], Chongqing [39], Finland [33], France [46,49], Iran [22], Ireland [32], Italy [51,53], Japan [36,37], Russia [25], Singapore [21,24], Spain [7], Sweden [35], Switzerland [47], Turkey [38], UK [41], USA [27,44,52].

**Table 1:** Summary characteristic of included study.

Number	Author	Year (Implementation/published)	Country	Design	Sample size	The type of neuraxial analgesia	Exclusion of mental health	Outcome evaluation tool (s)	Age of participants (years)	Quality Assessment	Outcome evaluation period	Findings
1	Banovcinova et al.	2019	Slovakia	Cross-sectional	510	Epidural	No	EPDS	19–45	–	NR	“Epidural analgesia was not associated with postpartum depression.”
2	Barooti et al.	2015–2017	Iran	Prospective cohort	200	Epidural	Yes	EPDS	all ages	Medium	7 days and 4 weeks postpartum	“Use of regional anesthesia reduces the incidence of PPD in the 4th week.”
3	Deng et al.	2014–5	China	Prospective cohort	599	Epidural and spinal-epidural	Yes	EPDS	18–34	Medium	6 weeks postpartum	The incidence of postpartum depression was lower in parturients with neuraxial analgesia than in those without.
4	Ding et al.	2014	China	Prospective cohort	214	Epidural	Yes	EPDS	all age	Medium	3 days and 6 weeks postpartum	“Postpartum depression occurred in 14.0% of parturients who received LEA and in 34.6% of those who did not.
5	Eckerdal el al.	2009–17	Sweden	Cohort	1503	Epidural	No	EPDS	>18	Medium	6 weeks postpartum	“Epidural analgesia was associated with depressive syndrome at 6 weeks postpartum.”
6	Edipoglu et al.	2020	Turkey	Prospective cohort	92	Epidural	Yes	EPDS	18–45	Medium	6 weeks postpartum	“There was a significant difference between both groups in terms of EPDS scores.”
7	Floris et al.	2006–7	Switzerland	Prospective cohort	79	Epidural	No	STAI	all age	Medium	2 h and 4 months postpartum	“Postnatal anxiety was not related to LEA or pain.”
8	Gaillard et al.	2007–9	France	Prospective cohort	264	Epidural	No	EPDS	≥16	Medium	6–8 weeks postpartum	There was no significant correlation between epidural and postpartum depression.
9	Hernández-Martínez el al.	2019	Spain	Cross-sectional	1531	Epidural or General anesthesia	NR	PPQ	≥18	High	1 and 5 years postpartum	“Use of epidural analgesia was protective factor against PTSD.”
10	Hiltunen et al.	2004	Finland	Prospective cohort	185	Epidural /Paracervical	NR	EPDS	19–44	Medium	2–7 days and 4 months postpartum	“The adjusted risk of depressive scores at the first postnatal week was decreased in the epidural/paracervical group when compared with no analgesia group.”
11	Johnstone al.	1995–6	Australia	Prospective	490	Epidural anesthesia	Yes	EPDS	16.0–42.8	Medium	8 weeks postpartum	“There was an increased but statistically non-significant risk of developing PND for epidural anesthetic.”
12	Kountanis el al.	2016–17	USA	Prospective cohort	600	Epidural	Yes	EPDS, PC-PTSD	≥18	Medium	3 months and 1 year postpartum	The association between severe pain despite neuraxial analgesia or anesthesia and PPD or PTSD was not statistically significant.
13	Lim et al.	2020	USA	Prospective cohort	72	Epidural	No	EPDS, STAI, PSS	≥18	Medium	6 weeks and 3 months postpartum	There was significant correlation between epidural and anxiety-state and anxiety-trait. However, there was not statistically significant correlation between epidural and perceived stress.
14	Lim et al.	2018	USA	Retrospective cohort	201	Epidural	No	EPDS	all age	High	6 weeks postpartum	Women with higher improvements in pain were associated with lower EPDS scores (r = 0.025, P = 0.002).
15	Liu el al.	2014–7	China	Prospective longitudinal	508	Neuraxial labour analgesia	Yes	EPDS	28–32	Medium	2 years postpartum	“The use of neuraxial analgesia during labour was associated with a significantly decreased risk of 2 year depression.”
16	Lyons	2016	UK	Prospective	64	Epidural analgesia	NR	IES, EPDS	18–39	–	1 month	“Women who were given an epidural were found to report more symptoms of post-traumatic stress.”
17	Munro el al.	2015–19	Canada	Prospective cohort	909	Epidural	No	EPDS	≥18	High	3, 6, and 12 months postpartum	There was no significant relationship between LEA and depression.”
18	Nagle el al.	2019–20	Ireland	Cross-sectional	1154	Spinal/Epidural General anesthetic	No	EPDS CBiTS	all age	Medium	6–12 weeks postpartum	“There was no significant relationship between Spinal/Epidural general anesthetic and traumatic birth.”
19	Nahirney el al.	2010	Canada	Prospective cohort	206	Epidural analgesia	Yes	EPDS	≥18	Medium	6 weeks and 6 months postpartum	“We found an incidence of PPD of 13.3% and no statistically significant association between epidural use and PPD.”
20	Ponti el al.	2014	Italy	longitudinal	186	Epidural analgesia	Yes	EPDS	≥18		1 month postpartum	“Anxiety and depression were positively linked with epidural.”
21	Ren el al.	2019	China	Prospective cohort	198	Epidural anesthesia	Yes	EPDS	18–45	Medium	6 weeks postpartum	“Epidural labor analgesia, family income and EPDS scores in the early postpartum period were the independent predictors of PPD.”
22	Riazanova el al.	2018	Russia	Prospective observational	210	Epidural analgesia	Yes	EPDS	26–32	Medium	6 h, 3 days and 6 weeks postpartum	“The use of epidural analgesia leads to a significant reduction of stress response during natural delivery, increases the risk of baby blues in the early postnatal period, but slightly influences the frequency of postpartum depression.”
23	Shishido el al.	2020	Japan	Longitudinal Observational	65	Epidural Analgesia	Yes	STAI, Stein's MB scale	20–40	Medium	1-2 and 4–5 days postpartum	There was no significant relationship between anxiety, maternity blues score at 1–2 days postpartum and epidural analgesia. However, there was significant relationship between maternity blues score at 4–5 days postpartum and epidural.”
24	Smorti el al.	2019	Italy	Longitudinal cohort	161	Epidural analgesia	Yes	STAI, EPDS	18–42	High	1 month postpartum	“The level of PPD is positively and significantly correlated with the duration of the administration of epidural analgesia.”
25	Suchanecki el al.	2022	France	Longitudinal cohort	100	Epidural anesthesia	NR	TES for PTSD, EPDS	18–41	Medium	NR	“There was no significant relationship between planning an epidural and traumatic event.”
26	Suhitharan el al.	2010–13	Singapore	Case–control	62 cases of PPD and 417 controls	Epidural analgesia	No	EPDS	19–48	Medium	4–8 weeks postpartum	“The absence of labor epidural analgesia remained as an independent risk factor for development of PPD when adjusted for psychiatric predictors of PPD such as history of depression or PPD and family history of depression.”
27	Sun el al.	2017–8	China	Prospective cohort	423	Epidural analgesia	No	EPDS	all age	Medium	48 h and 42 days postpartum	“It was found that epidural analgesia during labor may be associated with a decreased risk of PPD.”
28	Suzumori el al.	2011–14	Japan	Prospective cohort	104,065	Epidural analgesia	No	EPDS	all age	Medium	1, 6 and 12 months postpartum	“Risk of postpartum depression at six months after childbirth tended to be increased after vaginal delivery with anesthesia, compared with vaginal delivery without analgesia.”
29	Tan el al.	2009–15	Singapore	Prospective cohort	651	Epidural analgesia	NR	EPDS, STAI	all age	High	3 months postpartum	“There was no significant difference between women who received labour epidural analgesia and those who did not receive epidural analgesia in the incidence of PPD 3 months postdelivery.”
30	Tobin el al.	2017	USA	Prospective cohort	65	Epidural Analgesia	No	EPDS	all age	Medium	6–8 weeks postpartum	“Labor epidural analgesia did not reduce the risk of postpartum depression.”
31	Wu el al.	2006–2012	Canada	Population- based matched cohort	40,303	Epidural analgesia	NR	Yes	18–49	High	12 months postpartum	“Intrapartum epidural use was not associated with maternal postpartum depression.”

NR= Not Report; LEA = Labor Epidural Analgesia; EPDS = Edinburgh Postnatal Depression Scale; PPD=Postpartum Depression; STAI= State-Trait Anxiety Inventory; LEA = lumbar epidural analgesia; PTSD= Post Traumatic Stress Disorder; PPQ= Perinatal Posttraumatic-stress Questionnaire; PND= Postnatal Depression; PSS = perceived social support; IES= Impact of Event Scale.

Among the existing studies, 13 studies reported PPD results as a percentage [21,22,24,25,[33], [34], [35], [36],^39^,^40^,^50^,^52^,^54^], four studies reported PPD results as mean (Standard Deviation) [38,42,43,48], and three studies reported PPD results as odds ratio [26,39,44]. The results related to anxiety were reported in three studies [27,37,47]. One study reported PTSD as mean (SD) [49], three studies as a percentage [7,32,44], and one study reported only a significant level [41]. Nine studies had high-quality and 22 studies had intermediate quality according to the Newcastle-Ottawa scale (Table 1).

### 3.2 Meta-analyses results related to PPD

**Fig. 2:**
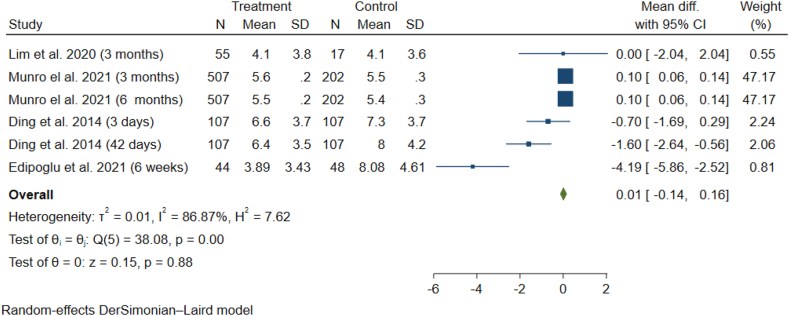
Meta-analysis of mean (SD) of postpartum depression in case and control studies.

According to the meta-analysis of the results of Edipoghlu et al. Lim et al., Munro et al. and Ding et al. studies using the random effect model, there is no statistically significant difference between the exposed (women receiving epidural) and non-exposed groups (no epidural) regarding the mean depression score (Mean Difference = 0.01; 95% Confidence Interval = −0.14 to 0.16; p-value = 0.88) with a considerable level of heterogeneity (I-squared = 86.8%, p-value<0.01) (Fig. 2). Also, the results of Egger's test indicated the publication bias among the studies (p-value< 0.001).

**Fig. 3:**
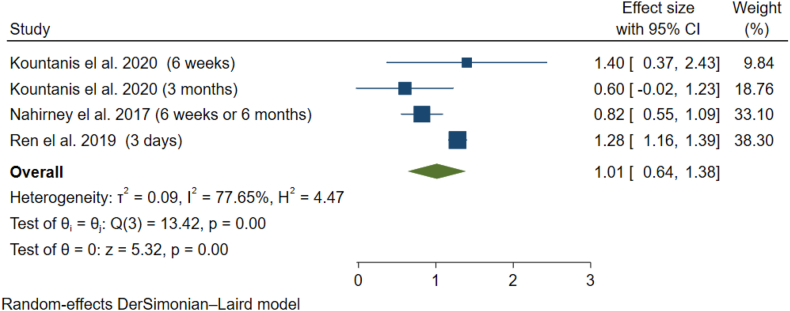
Meta-analysis of odds ratio of postpartum depression in case and control studies.

The meta-analysis of studies by Barooti et al. Deng et al., Eckerdal et al. Hiltunen et al., Riazanova et al. Sun et al., Suzumori et al. Tan et al., Tobin et al. and Ren et al. using a random effect model indicate a lower chance of depression in the epidural group compared to the non-epidural group, but this reduction was not statistically significant (OR = −0.61; 95% CI = −1.48 to 0.26; p-value = 0.17). Due to the high heterogeneity between the studies (I-squared = 99.7%, p-value <0.01), subgroup analysis was conducted on sample size [<500: (OR = 0.19; 95% CI = −1.08 to 0.70), heterogeneity (I-squared = 86.8%, p-value< 0.001); ≥ 500: OR = 0.61; 95% CI = −1.48 to 0.26), heterogeneity (I-squared = 86.8%, p-value< 0.01)] and follow-up time [<6 weeks: OR = 1.19; 95% CI = 1.08 to 0.70), heterogeneity (I-squared = 98.8%, p-value< 0.01); ≥ 6 weeks: OR = 0.61; 95% CI = −2.18 to 1.66), heterogeneity (I-squared = 99.8%, p-value <0.01)], however it did not resolve the heterogeneity (Fig. 3). According to Egger's test, publication bias was observed between the studies (p-value = 0.04). The Trill and Fill method was used to eliminate the publication bias. In this method, by adding 4 studies to the total of existing studies, the chance of depression in the exposed group was higher than in the non-exposed group (OR = 1.19; 95% CI = -1.96 to 0.41).

**Fig. 4:**
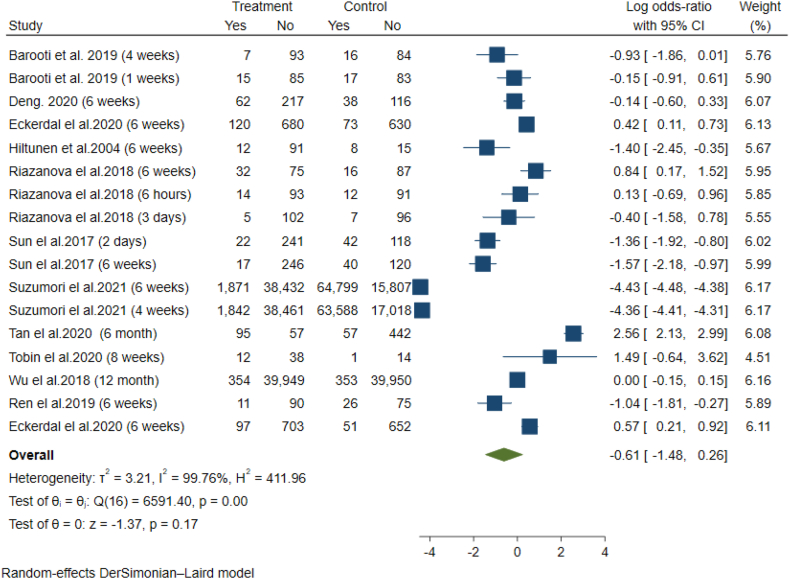
Meta-analysis of percentage of postpartum depression in case and control studies.

The meta-analysis of the studies by Kountanis et al. Nahirney et al., and Ren et al. using the random effect model (I-squared = 77.6%, p-value<0.01) showed a statistically significant difference in terms of the chance of depression between the exposed and non-exposed groups (OR = 1.01; 95% CI = 0.64 to 1.38; P-value = 0.00) (Fig. 4). Also, the results of Egger's test indicate the absence of publication bias in the study results (p-value = 0.79).

A longitudinal cohort study by Smorti et al. [51] investigating the risk factors associated with postpartum depression has shown a positive and significant relationship between the duration of epidural analgesia administration and postpartum depression (MD = 1.87; 95% CI = 0.48 to 1.14; p-value = 0.00). A longitudinal study by Liu et al. [50] into the relationship between epidural analgesia and the reduction of the risk of depression in mothers two years after birth showed that the rate of postpartum depression was significantly lower in the group exposed to epidural analgesia than in the non-exposed group (7.3% vs. 13.6%; p-value = 0.029). A cohort study by Lim et al. [48] found a statistically significant relationship between epidural analgesia and the reduction of postpartum depression (OR = 6.6; 95% CI = 1.9 to 22.4; p-value = 0.003). A cross-sectional study by Banovcinova et al. [23] to determine the predictors of postpartum depression showed that epidural analgesia is not one of the predictors of postpartum depression (β = −0.03; 95% CI = −1.35 to 0.57; p-value = 0.425). A case-control study by Gailard et al. [46] evaluated the relationship between various psychological, midwifery, and individual-social factors with postpartum depression and found no significant difference between the case group (with postpartum depression) and the control group (without postpartum depression) regarding the use of epidural analgesia (OR = 1.1; 95% CI = 0.4 to 2.6; p-value> 0.05). The study by Johnstone et al. [45] investigated the relationship between obstetric risk factors and postpartum depression and found that epidural analgesia did not increase the risk of postpartum depression (OR = 1.36; p-value>0.05). The study by Suhitharan et al. [21] investigated the relationship between analgesic and psychological factors and risk of postpartum depression and found that the risk of postpartum depression was significantly lower in women who received epidural analgesia (33 of 329 [10.0%]) than those who did not (29 of 150 [19.3%]) (P = 0.0078).

### 3.3 PTSD

Cohort studies by Kountanis et al. [44] and Suchanecki et al. [49] showed no statistically significant relationship between epidural analgesia and PTSD (OR = 0.83, 95% CI = 0.29 to 2.40; p-value = 0.734) (Mean score = 39.54; p-value = 0.050) respectively. In a prospective cohort study by Lyons et al. [41] it was shown that there was a statistically significant relationship between receiving epidural analgesia and PTSD symptoms (p-value< 0.02). A cross-sectional study by Hernández-Martínez et al. [7] found that epidural analgesia is a protective factor for PTSD symptoms (OR = 0.44; 95% CI = 0.24 to 0.80). In a cross-sectional study, Nagle et al. [32] showed that receiving epidural analgesia was not one of the predictive factors for PTSD (OR = 1.15; 95% CI = 0.85 to 1.55; p-value = 0.380).

### 3.4 Postpartum anxiety

**Fig. 5:**
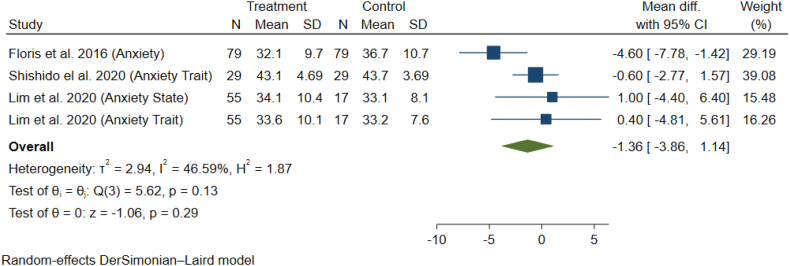
Meta-analysis of mean (SD) of anxiety in case and control studies.

The meta-analysis of studies by Floris et al. Shishido et al., and Lim et al. has shown that (I-squared = 46.5%, p-value = 0.13) the average anxiety score in the exposed group (women receiving epidural) was lower than the non-exposed group (not receiving epidural), however, it was not statistically significant (MD = −1.36; 95% CI = −3.38 to 1.14; p-value = 0.29) (Fig. 5). According to the results of Egger's test, no publication bias was observed between the studies (p-value = 0.53).

## 4 Discussion

This study is the first meta-analysis to investigate the association between receiving epidural analgesia and postpartum psychiatric disorders among women. According to the results of two different meta-analyses of depression studies, there was no statistically significant difference in the mean depression score between the exposed group (women receiving epidural) and the non-exposed group (women not receiving epidural). Another meta-analysis found a statistically significant difference between the exposed and non-exposed groups in terms of depression. There was no statistically significant association between epidural analgesia and PTSD in the cohort studies of Kountanis et al. Suchanecki et al., and Lyons et al. In addition, in two cross-sectional investigations of Hernández-Martnez et al. and Nagle et al. epidural analgesia had a conflicting influence in PTSD symptoms. In the Hernández-Martnez et al. it was a protective factor and in Negle et al. study epidural analgesia was not a predictive factor for the development of PTSD. Because of the variety in the methods used to measure PTSD and the heterogeneity in the methodology of the investigations, it was impossible to include the studies in a meta-analysis. In the meta-analysis of anxiety studies, the difference in mean anxiety score between the exposed and the non-exposed groups was not statistically significant.

PPD: According to the results of two different meta-analyses of depression studies, there was no statistically significant difference in the mean depression score between the exposed group (women receiving epidural) and the non-exposed group (women not receiving epidural). These results are consistent with the meta-analysis results of Parise et al. [28], Kountanis et al. [55], Almeida et al. [56], Zinger et al. [57], and another meta-analysis found a statistically significant difference between the exposed and non-exposed groups in terms of depression. The results were controversial due to the fact that the outcome of the PPD will most likely vary depending on the diagnostic criteria and follow-up duration used. For example, depression is most common one year after birth, however, most studies have a shorter follow-up time [28]. The follow-up duration in most studies ranged between 2 h and 8 weeks, with the exception of Hernández-Martnez et al.'s [7] and Kountanis et al.'s [44] studies, which had follow-up periods of 1 and 2 years, respectively. As a result, it can be stated that in most studies, the follow-up period was insufficient, and it is possible that this is why a significant statistical relationship between epidural analgesia and psychiatric disorders was not demonstrated. On the other hand, while the diagnosis of psychiatric disorders needs the approval of psychologist, in almost all studies, screening tools have been used as a diagnostic method [57]. Different cut-offs have been used to diagnose depression in different studies, which can impact the ability of the tool to detect depression. In the study by Edipoghlu et al. [38] depression is defined as a score of 10 or higher; in the study by Eckerdal et al. [35] a score of 12 or higher; and in the study by Hiltunen et al. [7] a score of 13 or higher.

Recently, epidural analgesia has been proposed as a possible modifiable factor for postpartum depression. This hypothesis is founded on the association between chronic pain and the onset of psychiatric disorders. Nevertheless, in this study the association between the use of epidural and postpartum depression was contradictory, the relationship between epidural analgesia and postpartum psychiatric disorders cannot be ruled out with absolute certainty [56].

PTSD: Due to the heterogeneity of the tools used to measure PTSD and the heterogeneity of the methodology of the studies, it was not possible to measure the studies in a meta-analysis. As a result, it is impossible to reach a definitive conclusion in this case. In addition, because the majority of the included studies were cross-sectional, it is impossible to accurately evaluate the cause-and-effect relationship in these studies. Longitudinal studies or randomized controlled clinical trials should be devised and conducted to more accurately assess the association between epidural analgesia and the incidence of PTSD [58].

Anxiety: In this study, there was no statistically significant difference between the anxiety score in the exposed group (women receiving an epidural) and the non-exposed group (women not receiving an epidural). These results are consistent with a longitudinl study by Shishido et al. [37]. Anxiety is a complex emotion and women during labour experience both external (contractions and pain) and internal (anticipation and imagination of future birth) component of anxiety. Furthermore, some confounders (provider approach, parity, family or professional support, history of depression, preterm birth, negative birth experience, and poor maternal self efficacy) [59] can affect postpartum anxiety [60].

Although the association between pain and mood disorders following childbirth has been hypothesized in individual studies [61], and pain relief may be linked to a decrease in these disorders [56], there is no statistically significant association between pain relief and psychological disorders in the current meta-analysis. This may be because of the following factors: 1. Compared to the control group, women who request epidural analgesia typically exhibit more feelings of fear, anxiety, and depression. These confounding and predisposing factors for psychological disorders can prevent the positive and protective effects of epidural analgesia on postpartum psychiatric disorders. 2. Research has shown that a number of various factors play a role in increasing risk of postpartum depression and anxiety [59], since postpartum psychiatric disorders are multifaceted. Pain was only one of the risk factors, and it is unlikely that only pain control with epidural analgesia is a protective factor for these disorders. 3. Every woman experience pain differently because it is a complicated sensation impacted by psychological and genetic variables. It's possible that using epidural analgesia does not alleviate pain or lessen postpartum psychiatric disorders in those with limited pain tolerance. The length of time before utilizing epidural analgesia has not been assessed in studies, which may be connected to how intense the labour pain is felt. In order to determine the association between pain relief and the decline in psychiatric problems, it is recommended that future studies look into the association between mothers' reported pain intensity and the length of time their pain went unrelieved [58]. 4. In China, for example, the prevalence of depression and other psychiatric disorders is higher than in other countries. It is challenging to achieve definitive conclusions when comparing women from different cultures who receive diverse emotional and psychological support from people around them. Furthermore, the results may differ based on the study location [57].

It is recommended that future studies aim for greater consistency by implementing longer follow-up periods, utilizing confirmation of psychiatric disorder diagnoses by qualified professionals, and employing standardized screening tools with standard cut-off points. Additionally, conducting randomized clinical trials could provide more conclusive results [28].

## 5 Conclusion

Although epidural analgesia is the gold-standard technique for reducing labour pain, it is controversial whether using epidural analgesia is useful in preventing postpartum psychiatric disorders or not. Since they are complex and affected by various factors. It may be beneficial to consider conducting studies with longer follow-up periods, utilizing the expertise of psychologists or psychiatrists for the diagnosis of psychiatric disorders, standardized screening tools, and conducting randomized clinical trials in order to obtain more conclusive results.

### 5.1 Strengths and limitations

The individual studies included in this meta-analysis also have several strengths. These include appropriate eligibility criteria and using a valid screening tool to detect PPD, PTSD, and anxiety. Anyway, there were several limitations in this study: **1**) Most of the studies were observational studies because of ethical concerns. Observational studies cannot account for unknown confounders that can only be adequately managed by randomized clinical trials. Due to the fact that most studies were not RCTs, the decision to perform neuraxial epidural analgesia may be affected by multiple factors, such as socioeconomic factors, which are important risk factors of PPD. **2**) Because of the limited number of observational studies, we could not be able to perform subgroup meta-analysis by study design. **3**) Even if all studies used Edinburgh Postnatal Depression Scale (EPDS) as a screening tool, the cut-off values of EPDS differed between studies. In some studies, the lack of standardization of the screening cut-offs may over- or underestimate the effect of neuraxial analgesia on PPD. **4**) EPDS is a highly validated screening tool, not a diagnostic instrument. The diagnosis of PPD may need confirmation by a psychiatrist. **5**) There is no universally accepted evaluation time point for PPD. Most studies screen PPD within 6 weeks after postpartum. However, approximately 7%–21% of depressive symptoms may last more than two years [62]. It is recommended that future studies include long-term follow-ups. Sixth, other risk factors of PPD, including adverse life events, parity, and breastfeeding, were underlying confounders that may not be adjustable.

## Ethics approval

The study has been approved by the Ethics Committee of Tabriz University of Medical Sciences, Tabriz, Iran (Code: IR. TBZMED.REC.1401.742).

## Consent for publication

Not applicable.

## Data availability statement

Data included in article/supplementary material/referenced in article.

## Funding

The study was funded by Tabriz University of Medical Sciences, Tabriz, Iran (Code: 70797).

## CRediT authorship contribution statement

**Solmaz Ghanbari-Homaie:** Writing – review & editing, Writing – original draft, Supervision, Methodology, Formal analysis, Data curation, Conceptualization. **Seyedeh-Pooneh Jenani:** Writing – review & editing, Writing – original draft, Visualization, Data curation. **Leili Faraji-Gavgani:** Software, Methodology, Formal analysis, Data curation. **Pooya Hosenzadeh:** Visualization, Resources, Conceptualization. **Mansour Rezaei:** Writing – original draft, Methodology, Funding acquisition.

## Declaration of competing interest

The authors declare that they have no known competing financial interests or personal relationships that could have appeared to influence the work reported in this paper.
