# Supplementary material for: Association between epidural analgesia and postpartum psychiatric disorders: A meta-analysis
Source: Heliyon. 2024 Mar 8;10(6):e27717. doi: 10.1016/j.heliyon.2024.e27717 (PMC10958359; doi:10.1016/j.heliyon.2024.e27717)
Supplement: Multimedia component 1 [file mmc1.docx]

Supplementary 1: The list of abbriviations of the article

| **Abbreviation** | **Complete form** |
| --- | --- |
| PPD | Postpartum Depression |
| PTSD | Post Traumatic Stress Disorder |
| STATA | Statistical Software for Data Science |
| EPDS | Edinburgh Postnatal Depression Scale |
| PRISMA | Preferred Reporting Items for Systematic and Meta Analysis |
| STAI | State-Trait Anxiety Inventory |
| PSS | Percieved Stress Scale |
| MB scale | Maternal Blues Scale |
| PPQ | Perinatal Post Traumatic Stress Questionnaire |
| PC-PTSD | Primary Care Post Traumatic Stress Disorder screening |
| CBiTS | City Birth Trauma Scale |
| TES | Traumatic Event Scale |
| IES | Impact of Events Scale |
